# Relationship between Decimal Hill Coefficient, Intermediate Processes, and Mesoscopic Fluctuations in Gene Expression

**DOI:** 10.1021/acsomega.4c09418

**Published:** 2025-04-01

**Authors:** Manuel Eduardo Hernández-García, Jorge Velázquez-Castro

**Affiliations:** Benemérita Universidad Autónoma de Puebla, Facultad de Ciencias Físico-Matemáticas, Avenida San Claudio y 18 Sur, Col. San Manuel, Heroica Puebla de Zaragoza, Puebla 72570, México

## Abstract

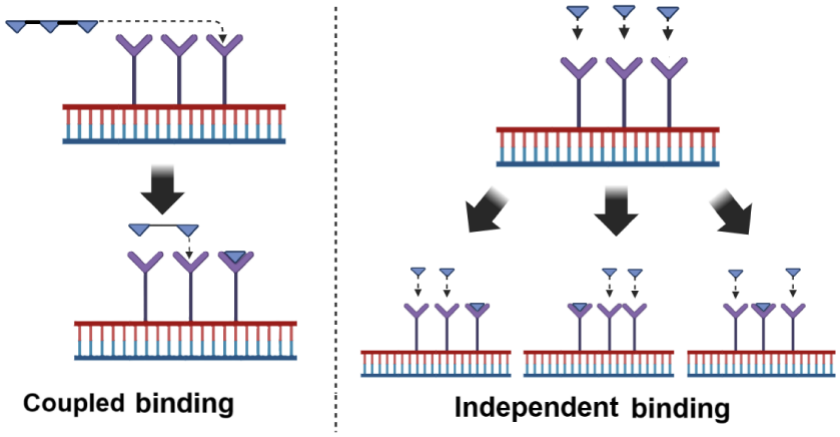

The Hill function is relevant for describing enzyme binding and other processes in gene regulatory networks. Despite its theoretical foundation, based on the mechanism of ligand–receptor binding, it is often used as a proper fitting function with a noninteger Hill coefficient in the description of gene expression. In this study, we explicitly considered intermediate processes in the transcription factor binding sites and mesoscopic concentration fluctuations, which, in contrast to the case of a single binding site without conformal states or all-or-none binding, lead to a noninteger Hill coefficient for the transcription rate. The relationships between the intermediate processes and the decimal Hill coefficient were established through a direct relationship between the dissociation constants, both with and without fluctuations. This outcome contributes to a deeper understanding of the underlying processes associated with the decimal Hill coefficient of gene expression rates while also enabling the prediction of an effective value of the Hill coefficient from the underlying mechanism. This procedure provides a simplified and effective description of the complex mechanisms that underlie gene expression.

## Introduction

The Hill function plays an important role in describing enzyme binding, as well as in the description of promoter activity in gene regulation networks.^1,2^ However, even if an underlying theory exists for its derivation based on ligand–receptor binding, it is sometimes used as an empirical description of a more complex biochemical process in the description of gene expression. As is the case with the Hill coefficient,^3^ which is commonly represented as *n*, this coefficient describes cooperativity in the binding of ligands to a receptor molecule, such as transcription factors to binding sites or the binding of metabolites to a transcription factor that activates it. In kinetic experiments, data are commonly fitted to a Hill function, and a decimal value is assigned to the Hill coefficient, even if single-site promoter models suggest integer values. Historically, this is similar to the results of a study on the interaction between hemoglobin and oxygen,^4^ where the Hill coefficient was found to be *n* = 1.8–3.4. However, hemoglobin can bind up to four oxygen molecules,^4,5^ and it was expected that the value of *n* should be equal to four. This process is more intricate than ligand–receptor binding, as described by Hill. In,^6,7^ intermediate processes were used to provide a more realistic representation of enzymatic reactions, given that ligands do not always bind simultaneously to a receptor, illustrating how various system parameters can produce different curves with varying degrees of cooperation. On the other hand, the relationship between the parameters of a Hill function describing gene expression of genes with more than one transcription factor binding site and the corresponding binding process remains unexplored.^8,9^ Although, insights can be gained from studies of less complex enzymatic processes.^10,11^

This study seeks to show that using a Hill function with decimal coefficients in gene expression entails the presence of intermediate processes and that the Hill coefficient corresponds to the coefficients of the detailed description that includes the intermediate processes.^7^

On the other hand, it is widely recognized that fluctuations can affect the dynamics of genetic regulatory networks.^12,13^ Although deterministic models can effectively capture most system dynamics,^1,9,12^ they are insufficient for explaining all the observed behaviors.^14^ The effect of intrinsic fluctuations on sigmoidal responses has already been studied, revealing corrections introduced by covariances in Michaelis–Menten dynamics.^15−18^ Using a similar approach, we also investigated the influence of intrinsic fluctuations in transcription factor concentrations on genes with multiple promoter-binding sites. It is worth noting that the resulting kinetic is equivalent to a single site in the promoter region where an activated transcription factor requires multiple metabolites for its activation.

We employed a systematic expansion around deterministic behavior to derive the mesoscopic corrections.^15,16,19,20^ Subsequently, we established relationships between the intermediate processes and the decimal Hill coefficient, both with and without fluctuations. Developing Hill functions with stochastic corrections for intermediate processes can improve the quantification of fluctuations in such systems.

In Section 2, we offer a concise overview of the mesoscopic approximation used to study the ligand–receptor binding processes that lead to the derivation of the Hill function. Sections 3 and 4 briefly address the deterministic forms of the Hill function, with and without intermediate processes, respectively. Section 5 presents the derivation of the Hill function with intermediate processes and stochastic corrections. In Section 6, we establish a connection between the Hill function with a decimal coefficient and the Hill function with intermediate processes, with and without fluctuations. Finally, in Section 7, we present our findings and draw conclusions based on the results.

## Mesoscopic Approximation

It is generally accepted that stochastic models must eventually converge with deterministic models when dealing with large systems. Furthermore, stochastic methods should provide an estimate of the fluctuations in the system. A widely utilized approach is linear noise approximation, which expands the master equation in terms of a small parameter proportional to the reciprocal of the system size.^12,13,15,16^ An alternative approach is to expand directly around the mean values of the concentrations,^15^ thereby enabling a straightforward method for calculating the mesoscopic corrections to macroscopic dynamics.

Consider *N* species *S*_*j*_ (*j* ∈ {1,2···,*N*}), and *M* reactions _i_ (*i* ∈ {1,2,···,*M*}) such that the species are transformed as
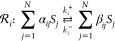
1

 and  are the reaction constants. The coefficients α_*ij*_ and β_*ij*_ are positive integers, from which we find the stoichiometric matrix
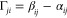
2

Through collisions (or interactions) of the different elements, they are transformed, following the mass kinetic law, so the macroscopic reaction rates as follows^13^
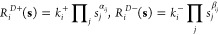
3where *s*_*j*_ is the mean concentration of the chemical species _j_,  is the variance of the concentration between the two chemical species  and , and **s**= (*s*_1_,*s*_2_,···,*s*_*N*_). The dynamical equation of the mean concentration is given by
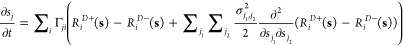
4we omit to write the equation of the dynamical evolution of  because we do not use this. For more detail about this equation and its derivation, please check,^19,20^ or the Supporting Information.

Based on (4), we can make the following substitution to obtain a mesoscopic correction to expressions involving deterministic reaction rates,
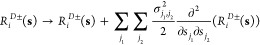
5

Notably, the correction term is proportional to the concentrations’ covariance and vanishes at the limits of large systems.

## Hill Function with Integer Hill Coefficient

Hill functions are commonly used to describe enzymatic reactions^21^ or to capture the dynamics of mRNA synthesis in gene regulatory circuits^1^ driven by transcription factors. Specifically, the Hill function is employed to describe the stationary concentration of a reversible process,^4^ as depicted by the following equation
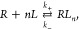
6where *n* are the required ligands *L* to be bound to the receptor *R* to activate the gene or enzyme. In gene activation, the ligands are transcription factors. The concentration of the product *RL*_*n*_ is dependent on the ligands concentrations *l* and is determined by a Hill function, as expressed by the following eq 7
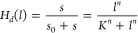
7where *s*_0_ is the concentration of the receptor *R* and *s* the concentration of *RL*_*n*_.  is the dissociation constant and *n* denotes the Hill coefficient.

**Figure 1:**
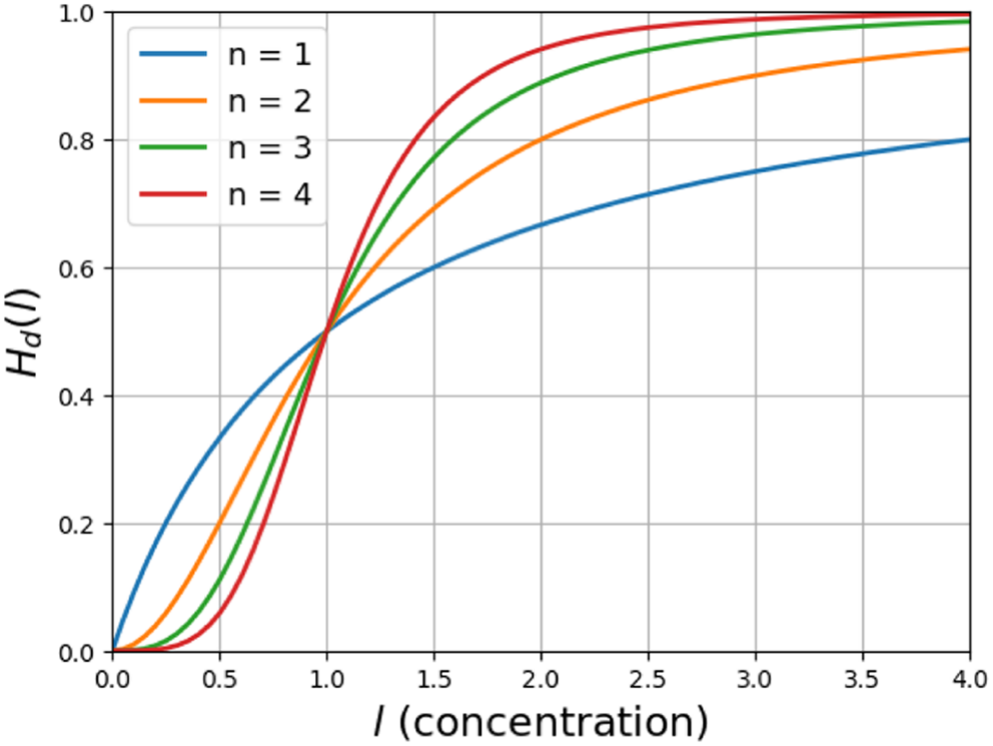
Deterministic Hill function. This figure shows the behavior of the deterministic Hill function for different values of *n* and *K* = 1.

Figure 1 depicts the Hill function behavior for various values of *n*.

## Ligand–Receptor Reactions with Intermediate Processes

Although Hill derived an equation for integer values of *n*, experimental data demonstrated that the coefficient can assume noninteger values. The interaction of hemoglobin with oxygen exemplifies this,^4^ hemoglobin can bind up to four oxygen molecules,^4,5^ and it was expected that the value of *n* should be equal to four; however, the experimental findings indicate a value of *n = 1.8–3.4*. This suggests that Hill’s original perspective is insufficient.^3^ Alternative methods for ligand binding to proteins, such as placing intermediate processes in which coupled or independent binding occurs,^6,7^ should be considered. There are various degrees of interactions between ligands and even in an independent case, the binding is rarely simultaneous. However, in this work, two limit cases were analyzed: coupled and independent binding. In coupled binding, the binding of a ligand to one site on a receptor allows for the binding of a subsequent ligand to another site. This means that the binding events are completely dependent on the previous ones. In contrast, in independent binding, the binding of a ligand to one site on a receptor does not affect the binding of ligands to other sites. Each binding site operates independently of the other binding sites.

**Figure 2:**
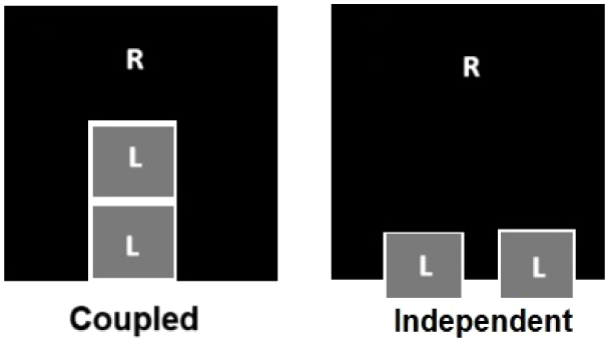
Schematization of coupled and independent ligands–receptor binding processes. In independent binding, the binding of a ligand to one site on a receptor does not affect the binding of ligands to other sites. Each binding site operates independently of other binding sites.

Figure 2 illustrates the idea of the cases considered in this article. It is not intended to represent a real physical-chemical process. Although there is a body of work on allosteric regulation in polymers^10^ in gene regulatory dynamics, models from enzymatic processes are directly applicable to a multisite binding promoter region.^1,7,8,22^

We focused on establishing a steady-state concentration of the product in ligand–receptor reactions involving intermediate processes. These intermediate processes can be either coupled or independent, as shown in Figure 2. The coupled binding scheme requires that the first binding site is filled in order for the second site to become occupied, for example, as if the ligand molecules stack on top of each other at their binding. In the independent binding scheme, two binding sites are available to the ligand; either one can be occupied independently of the other.^7^

### Coupled Case

In this scenario, only if the previous site is occupied can the next site be occupied (Figure 2), the following reactions occur, along with their corresponding reaction constants as reported in^7^
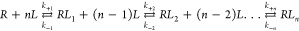
8where *n* represents the number of ligands (transcription factors) required to activate a gene (in this case). At the steady state, each chemical reaction must satisfy the equilibrium relationship given by the equation
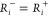
9

This relationship can be equivalently expressed as , where  and  are forward and backward reaction rates, respectively. In this case, the reaction rates are given by
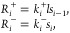
10

(*i* = {1, ..., *n*}), where we define *s*_0_ as the concentration of *R*, *l* as the concentration of *L* and *s*_*n*_ as the concentration of *RL*_*n*_. Using condition (9), a recurrent relation can be derived from the set of species *s*_*i*_’s, given by
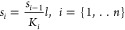
11

(). Solving for *s*_*i*_ in terms of *s*_0_ a relationship between them can be expressed as
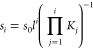
12

The Hill function, which represents the fraction of the product to all species in the reaction, is then expressed as
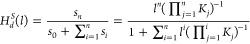
13

Because gene expression is activated or not by the relevant product, in the last equation, only the product of interest is in the nominator, even if the intermediates are also products.

In the case of several binding sites in the promoter region, all sites must be ligand-bound, which is a transcription factor that activates mRNA synthesis.^23^ Thus, defining a relevant product as an active gene is convenient.^7^ This convention contrasts with other definitions of the Hill function in which all intermediate states are considered products.^11^ For example, the exact fraction of product produced by a process of coupled ligands-receptor binding, for a case in which *K*_*j*_=*K* and *n* = 4 is given by
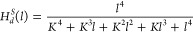
14

**Figure 3:**
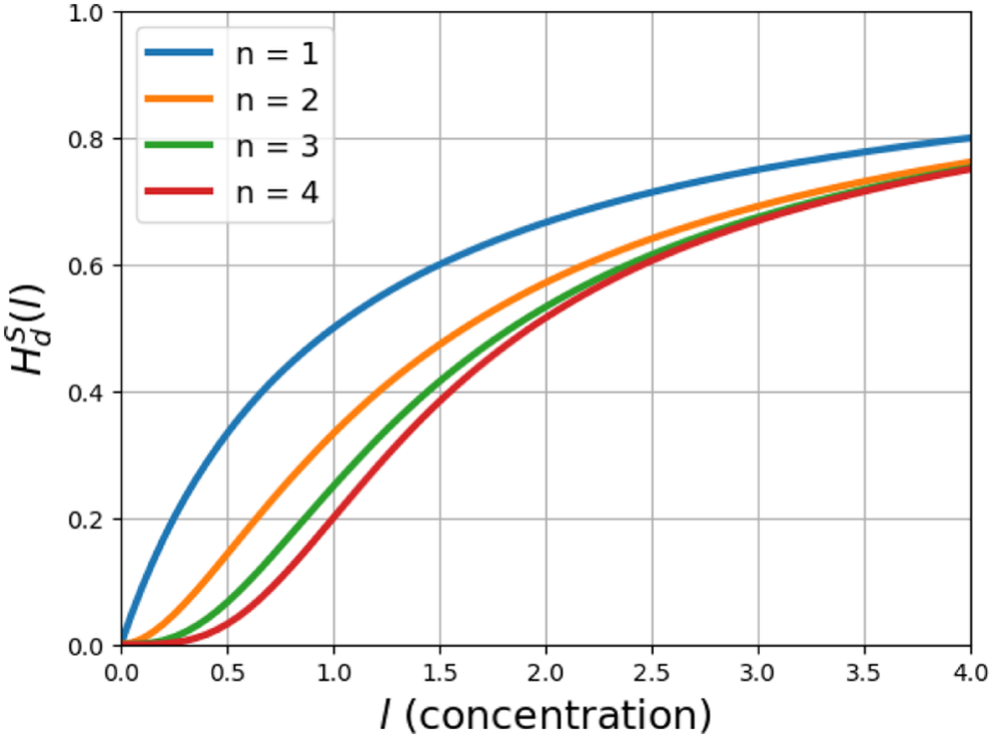
Hill function with coupled ligand–receptor binding. This figure shows the behavior of the Hill function where intermediate binding processes is coupled, with *K*_*j*_ = 1 and different values of *n*.

Figure 3 shows the behavior of function (13) for different values of *n*.

### Independent Case

In the case of independent binding, the following reactions and their reaction constants are considered^7^
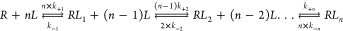
15where *n* represents the number of ligands (transcription factors) required to activate a gene (in this case). A similar analysis to the coupled case was performed but with the following reaction rates
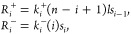
16

(*i*={1,···,n}), where *s*_0_ is the concentration of *R*, *l* is the concentration of *L* and *s*_*n*_ are the concentrations of *RL*_*n*_. The fraction of product in the reaction as a function of the ligand concentration is given by the next Hill function
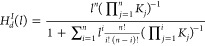
17

An interesting particular case is when *K*_*j*_ = *K*,
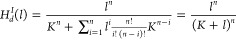
18

**Figure 4:**
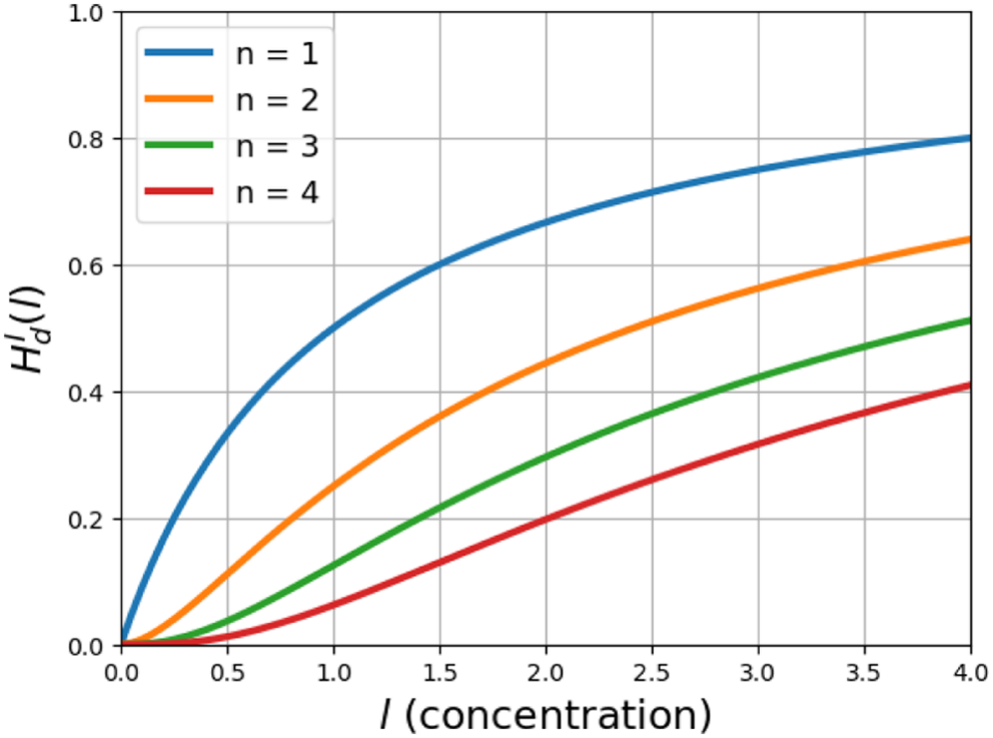
Hill function with independent ligand–receptor binding. This figure shows the behavior of the Hill function where intermediate binding processes occur independently, with *K*_*j*_ = 1 and different values of *n*.

Figure 4 shows the behavior of the function (18) for different values of *n*. As expected, when *n* = 1, the Hill function is recovered for both the coupled and independent cases. For the usual Hill function, the Hill coefficient can be better understood as a coefficient that describes the degree of interaction between different receptor sites, as shown in.^7^

## Intrinsic Fluctuations in Ligand–Receptor Reactions

In addition to the fact that ligands are not solely attached to receptors through a single process, which leads to an effective decimal Hill coefficient, inherent fluctuations can also affect the Hill coefficient value, causing it to deviate from integer values when we fit the experimental data. Intrinsic fluctuations might be important for low concentrations, such as proteins and transcription factors inside the cell.

### Coupled Case

In a ligand–receptor process in which coupled binding is required before the final product is produced, the following reactions apply,
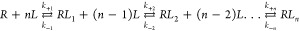


To account for inherent fluctuations, we utilize the expression (5), resulting in the following equations for  and ,
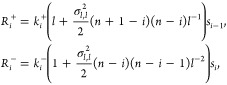
19where *i*={1,···,*n*}, *s*_0_ represents the concentration of *R*, *l* denotes the concentration of *L*, and *s*_*i*_ denotes the concentration of RL_*i*_. Stochastic corrections are included in these expressions based on the variance between the number of ligands by . As the reaction rates *R*_*i*_ depend on concentrations of both species, normally, their fluctuations are related to the fluctuations in the concentrations of the species and their correlations. However, we have assumed that species are only related by the explicit reactions taken into account thus their intrinsic fluctuations are independent. In particular, the fluctuations between *s*_*i*_ and *l* are assumed independent as in^16^ that is, .

In accordance with the stationary condition , we get
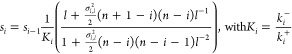
20

Solving for *s*_*i*_ in terms of *s*_0_, we find that
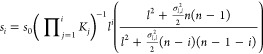
21and finally, the fraction of product in the reaction is given by
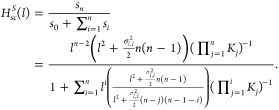
22

The last expression is the Hill-type function with stochastic corrections, which considers fluctuations in the concentrations of the ligands. In the case in which *n* = 1, a Michaelis–Menten-type function is recovered, and no corrections due to intrinsic fluctuations by ligands are observed, consistent with,^16^ where the stochastic corrections appear in the mean concentration of *R*. However, if *n* ≥ 2, corrections arise, induced by the varianc e of ligands *l*.

For the particular case of *n* = 4, *K*_j_=*K*, and ,^19^ where Ω = *N*_*A*_*V* is the size of the system and has units of *volume*/*mole*, and Avogadro’s number *N*_*A*_ is used to convert the number of the molecules to moles, and has units of 1/*mol*. This expression for σ^2^ is consistent with the fact that for large Ω, that is, large or macroscopic systems, the fluctuations in the concentration should vanish. We obtain
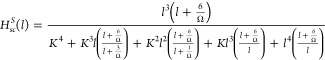
23

By comparing the last expression with its deterministic counterpart (14), it is evident that it differs significantly. Additionally, when Ω becomes very large, its deterministic counterpart is recovered.

**Figure 5:**
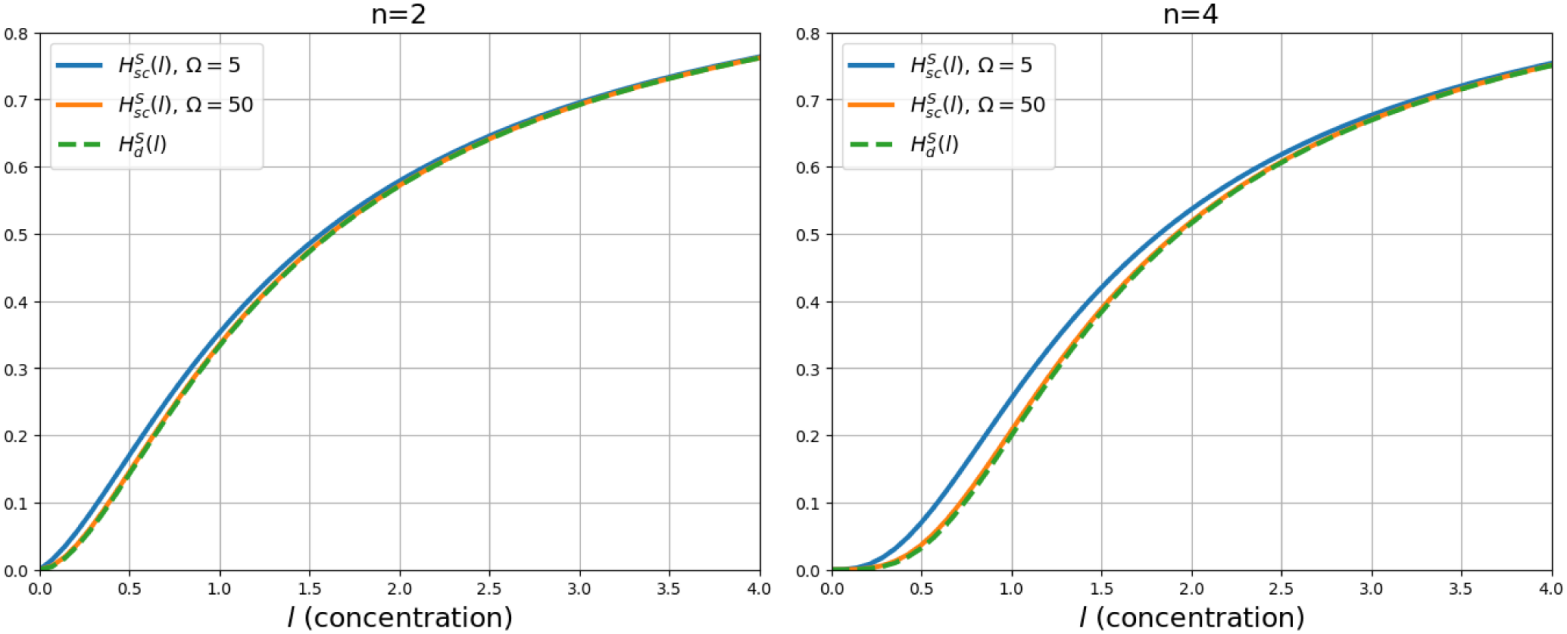
Hill function with coupled ligand–receptor binding. In this figure, we compare a Hill function with coupled intermediate processes in cases with and without intrinsic fluctuations. We observe that both functions are similar when Ω=50. We used *K*_*j*_ = 1 and two different values of *n*. (*l*) is the deterministic Hill function and (*l*) is the Hill function with stochastic corrections.

We present a plot of eq 22 for various values of *n* and *K*_*j*_=*K*. The results are shown in Figure 5. We compare the deterministic Hill function with intermediate processes  to one that includes stochastic corrections (*l*). We plot only two cases, for *n* = 2 and *n* = 4. The graph indicates that the two functions are very similar when Ω=50 in both cases. However, when Ω, there were slight differences between the two, particularly for low concentrations of *l*. Despite these differences, the graphs indicate that the two functions are practically equal when Ω is big enough.

### Independent Case

For the independent case, the Hill function with stochastic corrections can be obtained similar to the previous case, yielding the following expression
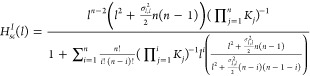
24

The last expression is a Hill-type function with stochastic corrections because it considers the fluctuations in the ligands. For the case in which *n* = 4, K_*j*_=*K* and , this expression reduces to
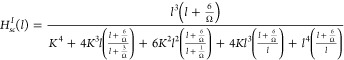
25

When Ω becomes large, its deterministic counterpart is recovered.

**Figure 6:**
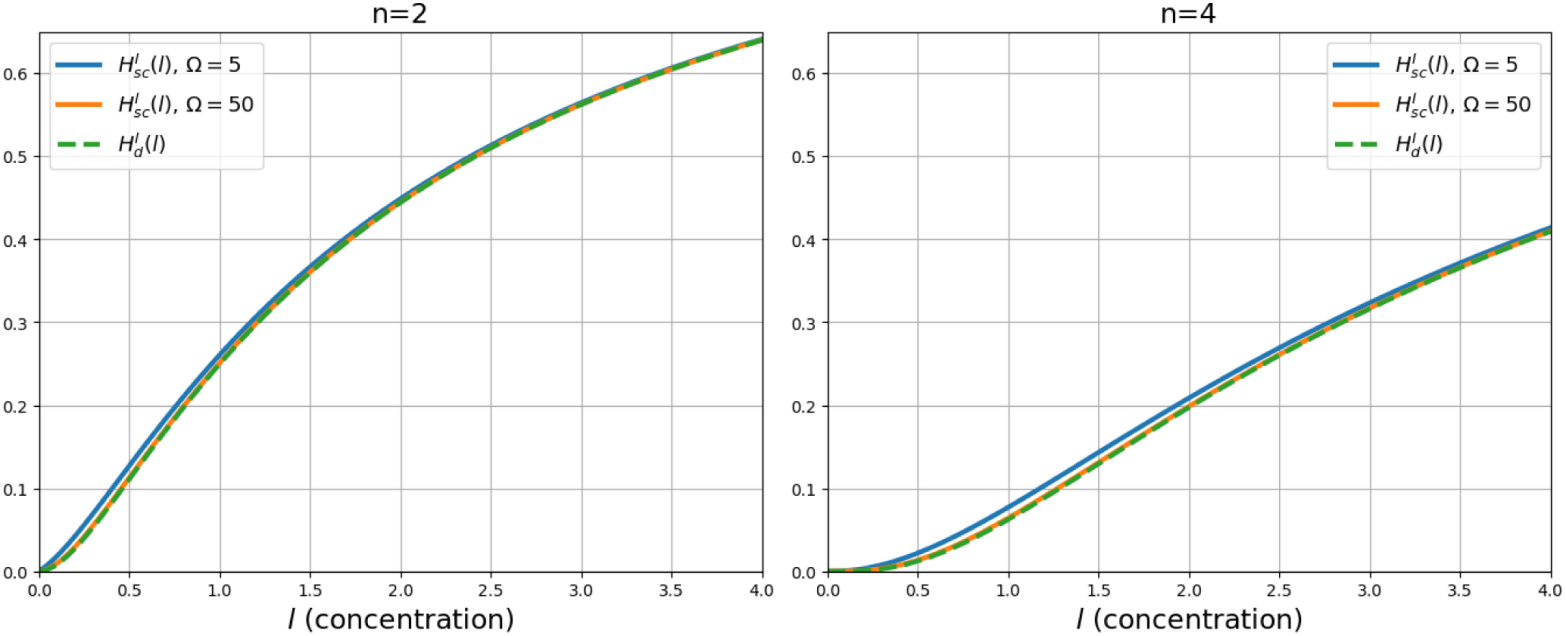
Hill function with ligand–receptor binding independent. In this figure, we compare a Hill function with independently occurring intermediate processes in the cases with and without intrinsic fluctuations. We can observe that both functions almost coincide. We used *K*_*j*_ = 1, Ω=5 and different values of *n*. With (*l*) the deterministic Hill function, and (*l*) the Hill function with stochastic corrections.

In Figure 6 we compare the deterministic Hill function with intermediate processes (*l*) and the Hill function with stochastic corrections (*l*). The figure reveals no discernible differences between the two Hill functions when Ω=50, suggesting that they are practically equivalent; however when Ω=5, there were few differences when the concentration of *l* was low.

For the two cases examined, stochastic corrections are introduced into the system as follows
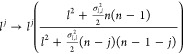
26this transformation represents a term that multiplies the ligand concentration and differs significantly when intermediate processes are not considered. This result differs from what was derived in^19^ because here, the stochastic corrections are introduced by the following expression,
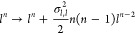
27where intermediate processes were not considered. This shows that when intermediate processes are considered in the Hill functions, how the corrections are made to the deterministic system differs, and eq 26 must be used.

The derived Hill-type functions, which incorporate intermediate processes and stochastic corrections, can be applied to gene regulation networks or to describe enzyme binding. Employing this type of Hill function offers more accurate results as it mitigates the risk of overestimation and enables more precise quantification of the inherent fluctuations within the system. When used in conjunction with the method described in^19^ or the Fluctuation Dissipation Theorem (FDT),^13^ it enhances the ability to comprehend and model natural variations in the system.

## Relationship between Intermediate Process and Hill Coefficient

This section presents a method for relating empirical Hill functions with decimal coefficients to coupled and independent reaction cases, with and without intrinsic fluctuations. A specific example is provided to illustrate how they can be related.

Consider a system where a maximum of *n*_*m*_ = 2 ligands can bind to a receptor. However, when fitting the experimental data, we obtained *n*_*e*_ (empirical decimal Hill coefficient) with 1<*n*_*e*_ < 2. By breaking down the Hill function (7), we obtain the following equation
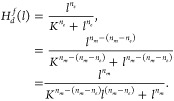
28

Next, we can expand  in power series around *l* = *K*. This choice was made because (*l*), (*l*) (deterministic Hill functions for coupled intermediate processes), and (*l*) (deterministic Hill functions for independent intermediate processes) have the same value at this point.^24^ Thus, we have
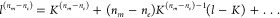
29if we substitute this series into (*l*) then it has a shape similar to the Hill function with an intermediate process. Substituting *n*_*m*_ = 2 the Hill function can be expressed as follows
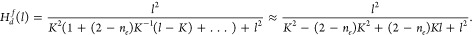
30

From this result, we can compare the values with the case when considering the intermediate processes. For *n*_*m*_ = 2, the deterministic Hill functions for coupled and independent intermediate processes are
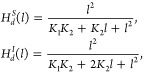
31

The first equation corresponds to coupled intermediate processes, and the second corresponds to independent intermediate processes. We observe a correspondence between the denominator values in (30) and (31). This correspondence is presented in Table 1.

**Table 1:** Relations Table without Fluctuations^a^

Relations for *n*_*m*_ = 2 and 1<*n*_*e*_ < 2		
Type	*K*_1_	*K*_2_
Coupled	*K*(*n*_e_-1)/(2-*n*_*e*_)	*K*(2-*n*_*e*_)
Independent	2*K*(*n*_*e*_-1)/(2-*n*_*e*_)	*K*(2-*n*_*e*_)/2

a Relation between the Parameter of (30) and (31) with the Deterministic Models.

Following a similar approach to that in,^10^ a relationship can be established between the decimal Hill coefficient and the system parameters for the case of coupled binding:
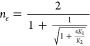
32and for independent binding
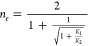
33

These results are similar to those obtained in polymer science.^10^ However, one important difference is that only the fully bound state is considered a product, as this is the activated state of a gene.

**Figure 7:**
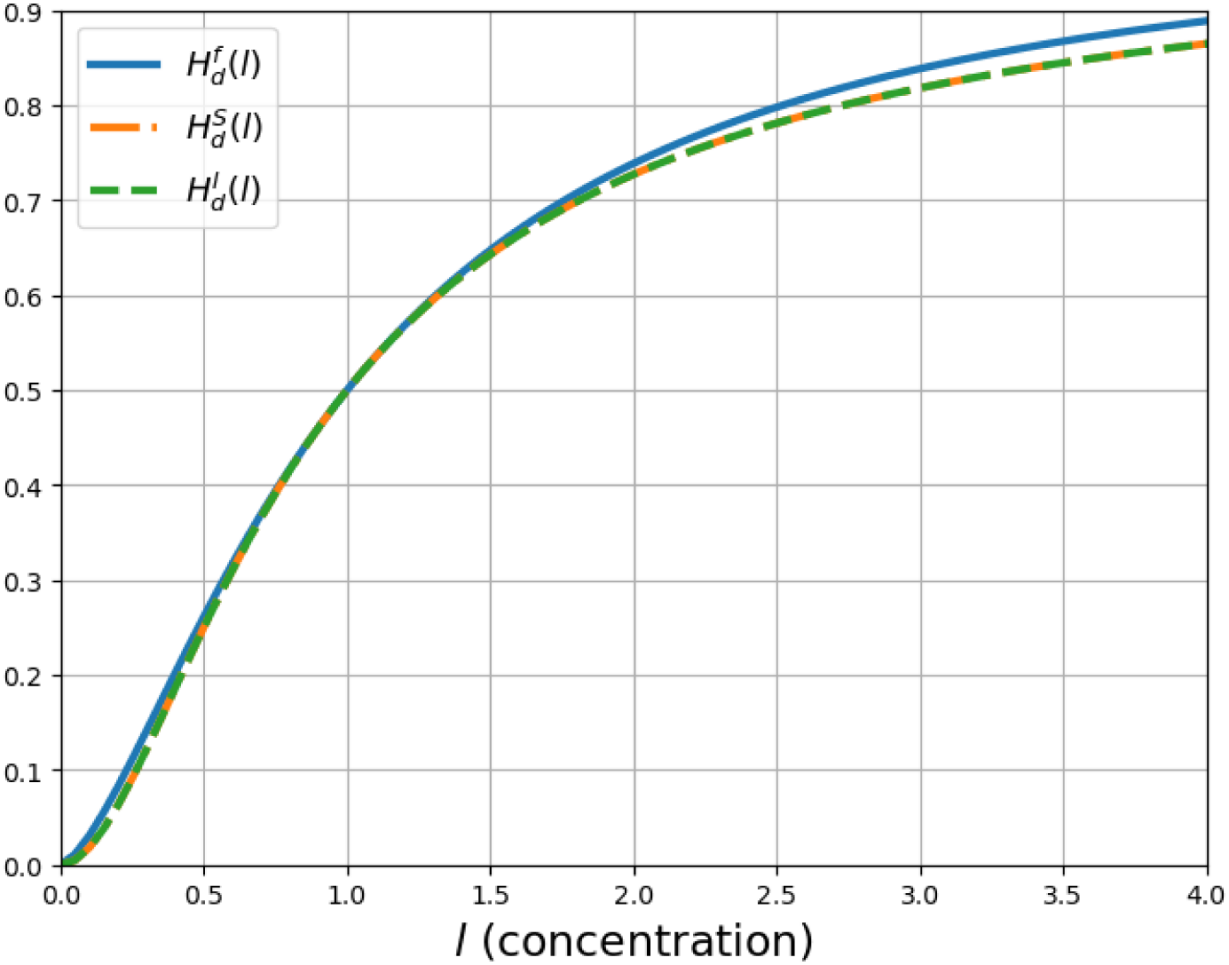
Comparison between deterministic models In this figure, we compare three deterministic models: one using a Hill function with a decimal coefficient ((*l*)), another with intermediate processes presented coupled ((*l*)), and the last one with processes presented independently ((*l*)). We observe that around *K* (*K* = 1), all three functions overlap. We used *K* = 1, *n*_*m*=2_, *n*_*e*=1.5_, and the values of the parameters in the functions are in Table 1.

Figure 7 showscoupled and independent cases as the concentration increases. When the concentrations are close to the value of *K* (*K* = 1), the graphs appear similar but diverge as the concentration increases. Despite the overlapping of the graphs, which makes it difficult to distinguish between coupled and independent cases in this system, it is important to note that fundamentally different mechanisms govern them.

From the examples provided, it can be concluded that a Hill function with decimal coefficients might involve intermediate processes in the reaction.

Now, we analyze the consequences of intrinsic fluctuations on the Hill coefficient. The Hill functions that involve intermediate processes with intrinsic fluctuations are taken from eqs 22 and 24. In the case *n*_*m*=2_, we get
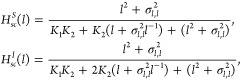
34where the first equation pertains to coupled intermediate processes and the second pertains to independent intermediate processes.

We use the rule derived in eq 26 to introduce fluctuations in a system with intermediate processes; this rule is
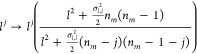
35

Substituting (35) into (30), results in
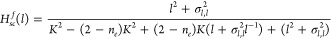
36

This is a Hill-type function with stochastic corrections that can be related to an empirical decimal Hill coefficient *n*_*e*_. The system parameters are recalculated by comparing (34) with (36). The results are presented in Table 2.

**Table 2:** Relations Table with Fluctuations^a^

Relations for *n*_*m*_ = 2 and 1<*n*_*e*_ < 2		
Type	*K*_1_	*K*_2_
Coupled	*K*(*n*_*e*_-1)/(2-*n*_*e*_)	*K*(2-*n*_*e*_)
Independent	2*K*(*n*_*e*_-1)/(2-*n*_*e*_)	*K*(2-*ne*)/2

a Relation between the Parameter of (34) and (36) Considering Intrinsic Fluctuations.

From the outcome of this analysis, it can be inferred that the parameters of the function (30), when accounting for stochastic corrections and employing expression (35), exhibit an exact alignment with the findings obtained in Table 1. In other words, the results are consistent with those obtained in the deterministic scenario. Consequently, eq 36 is suitable when considering intermediate processes with intrinsic fluctuations.

Thus, we can conclude that by introducing intrinsic fluctuations into the system, we can better adjust it using models with stochastic corrections and by changing the values of *n*_*e*_. Models with intermediate processes and stochastic corrections are more realistic because the ligands do not bind instantly to the receptors, and fluctuations are always present.

## Results and Discussion

A more general expression for the Hill function describing the transcription rates of gene regulatory networks can be obtained by considering intermediate and stochastic processes. In this study, we used two simple models of intermediate processes obtained from enzyme kinetics; however, more complex models can be proposed. For example, different allosteric mechanisms in polymers may have equivalent dynamics for some gene transcription processes. This kind of generalization of the Hill function can help improve the accuracy of the description of biochemical network dynamics when intermediate processes are present during gene activation and in the mesoscopic regime. In addition, the simplified description of complex processes by the Hill function can be helpful in simulating gene regulatory networks with a large number of nodes because it is computationally more efficient than the commonly used Gillespiee algorithm. Thus, the mesoscopic description opens the door to systems simulations with many components more closely related to real-world scenarios than other simplistic models.

By establishing a connection between the intermediate processes in gene activation and the Hill function with decimal coefficients, it was possible to show that the Hill function with a decimal coefficient is equivalent to describing a process with intermediate processes. Furthermore, when intrinsic fluctuations are introduced to the system, and a Hill function is parametrized with stochastic corrections, the decimal Hill coefficient varies from that obtained deterministically, showing that fluctuations can play an important role when calculating this coefficient. This relationship provides a better understanding of the underlying processes associated with the decimal Hill coefficient while also enabling the prediction of an effective value of the Hill coefficient from the underlying mechanism, allowing us to have a simplified description of the complex gene regulation process.

## Supporting Information Available

Additional detail to derive eq 4 using the moment-based approach into the Chemical master equation (PDF)
