# Supplementary material for: Relationship between Decimal Hill Coefficient, Intermediate Processes, and Mesoscopic Fluctuations in Gene Expression
Source: ACS Omega. 2025 Apr 1;10(14):13906–14. doi: 10.1021/acsomega.4c09418 (PMC12004157; doi:10.1021/acsomega.4c09418)
Supplement: Supplementary file 1 — ao4c09418_si_001.pdf [file ao4c09418_si_001.pdf]

# Supporting Information
# Relationship between Decimal Hill Coefficient, Intermediate Processes and Mesoscopic Fluctuations in Gene Expression

Manuel Eduardo Hernández-García* and Jorge Velázquez-Castro*

*Benemérita Universidad Autónoma de Puebla, Facultad de Ciencias Físico-Matemáticas, Avenida San Claudio y 18 Sur, Col. San Manuel, Heroica Puebla de Zaragoza 72570, Puebla, México.*

E-mail: manuel.hernandezgarcia@viep.com.mx; jorge.velazquezcastro@correo.buap.mx

Here, we derive Equation 4 of the principal text. Consider $N$ species $S_j$ ($j \in \{1, 2, \ldots, N\}$), and $M$ reactions $\mathcal{R}_i$ ($i \in \{1, 2, \ldots, M\}$) such that the species are transformed as

$$\mathcal{R}_i : \sum_{j=1}^{N} \alpha_{ij} S_j \underset{k_i^-}{\overset{k_i^+}{\rightleftarrows}} \sum_{j=1}^{N} \beta_{ij} S_j. \tag{1}$$

$k_i^+$ and $k_i^+$ are the reaction constants. The coefficients $\alpha_{ij}$ and $\beta_{ij}$ are positive integers, from which we find the stoichiometric matrix

$$\Gamma_{ji} = \beta_{ij} - \alpha_{ij}. \tag{2}$$

Through collisions (or interactions) of the different elements, they are transformed, so the propensity rates are given as follows[1]

$$t_i^+(\mathbf{S}) = k_i^+ \prod_j \frac{S_j!}{\Omega^{\alpha_{ij}}(S_j - \alpha_{ij})!}, \qquad t_i^-(\mathbf{S}) = k_i^- \prod_j \frac{S_j!}{\Omega^{\beta_{ij}}(S_j - \beta_{ij})!}, \tag{3}$$

where $\mathbf{S} = (S_1, S_2, ..., S_N)$, $\Omega$ is the size of the system and has units of volume per mole, with these propensity rates we have the next Chemical master equation:

$$\begin{aligned} \partial_t P(\mathbf{S}, t) = \Omega \sum_i \big( & t_i^+(\mathbf{S} - \Gamma_i) P(\mathbf{S} - \Gamma_i, t) - t_i^+(\mathbf{S}) P(\mathbf{S}, t) \\ & + t_i^-(\mathbf{S} + \Gamma_i) P(\mathbf{S} + \Gamma_i, t) - t_i^-(\mathbf{S}) P(\mathbf{S}, t) \big) \end{aligned} \tag{4}$$

The chemical master equation describes the temporal evolution of the system states. To derive the evolution of macroscopic quantities from the chemical master equation, a common approach involves multiplying the equation by the relevant quantities and then averaging. This procedure yields formal expressions for the equations governing the evolution of mean

concentrations, which can be written as follows:

$$\frac{\partial}{\partial t}\left(\frac{\langle S_j\rangle}{\Omega}\right) = \sum_i \Gamma_{ij}\left\langle t_i^+(\mathbf{S}) - t_i^-(\mathbf{S})\right\rangle. \tag{5}$$

The terms $\langle t_i^{\pm}(\mathbf{S})\rangle$ can be approximated using the following expansion around the mean:[2,3]

$$\begin{aligned}\langle f(\mathbf{X})\rangle \approx & \left\langle f(\langle \mathbf{X}\rangle) + \textstyle\sum_{j_1} (\langle X_{j_1}\rangle - X_{j_1}) \frac{\partial f(\mathbf{X})}{\partial X_{j_1}}\Big|_{\mathbf{X}=\langle \mathbf{X}\rangle} + \sum_{j_1}\sum_{j_2} \frac{(\langle X_{j_1}\rangle - X_{j_1})(\langle X_{j_2}\rangle - X_{j_2})}{2} \frac{\partial^2 f(\mathbf{X})}{\partial X_{j_1}\partial X_{j_2}}\Big|_{\mathbf{X}=\langle \mathbf{X}\rangle}\right\rangle \\ = & \quad f(\langle \mathbf{X}\rangle) + \textstyle\sum_{j_1}\sum_{j_2} \frac{\sigma^2_{j_1,j_2}}{2} \frac{\partial^2 f(\mathbf{X})}{\partial X_{j_1}\partial X_{j_2}}\Big|_{\mathbf{X}=\langle \mathbf{X}\rangle}. \end{aligned} \tag{6}$$

where $j_1, j_2 \in \{1, 2, \ldots, N\}$, and $\sigma^2_{j_1,j_2} = \langle(\langle X_{j_1}\rangle - X_{j_1})(\langle X_{j_2}\rangle - X_{j_2})\rangle$ is the covariance between variables $X_{j_1}$ and $X_{j_2}$. This approach is a second-order approximation of the mean, assuming that the fluctuations around the mean are small. The species mean concentrations are defined as $s_j = \frac{\langle S_j\rangle}{\Omega}$. Therefore, we can rewrite the mean concentration dynamics as

$$\frac{\partial s_j}{\partial t} = \textstyle\sum_i \Gamma_{ji}\left(R_i^{D+}(\mathbf{s}) - R_i^{D-}(\mathbf{s}) + \sum_{j_1}\sum_{j_2} \frac{\sigma^2_{j_1,j_2}}{2} \frac{\partial^2}{\partial s_{j_1}\partial s_{j_2}}(R_i^{D+}(\mathbf{s}) - R_i^{D-}(\mathbf{s}))\right), \tag{7}$$

where $\sigma^2_{j_1,j_2} = \frac{\langle(\langle S_{j_1}\rangle - S_{j_1})(\langle S_{j_2}\rangle - S_{j_2})\rangle}{\Omega^2}$, $\mathbf{s} = (s_1, s_2, ..., s_N)$, $R_i^{D+}(\mathbf{s})$ and $R_i^{D-}(\mathbf{s})$ are the deterministic or also called macroscopic reaction rates

$$R_i^{D+}(\mathbf{s}) = k_i^+ \textstyle\prod_j s_j^{\alpha_{ij}}, \quad R_i^{D-}(\mathbf{s}) = k_i^- \displaystyle\prod_j s_j^{\beta_{ij}}. \tag{8}$$

# References


(1) Gardiner, C. *Stochastic methods*; Springer Berlin, 2009; Vol. 4.

(2) Hernández-García, M. E.; Velázquez-Castro, J. Corrected Hill Function in Stochastic Gene Regulatory Networks. *arXiv preprint arXiv:2307.03057* **2023**,

(3) Gomez-Uribe, C. A.; Verghese, G. C. Mass fluctuation kinetics: Capturing stochastic effects in systems of chemical reactions through coupled mean-variance computations. *The Journal of Chemical Physics* **2007**, *126*.
